# Biodiesel production and simultaneous treatment of domestic and livestock wastewater using indigenous microalgae, *Chlorella sorokiniana* JD1-1

**DOI:** 10.1038/s41598-023-42453-y

**Published:** 2023-09-14

**Authors:** Jae-Cheol Lee, Kira Moon, Nakyeong Lee, Sangdon Ryu, Seung Hui Song, Yun Ji Kim, Sung Moon Lee, Hyun-Woo Kim, Jae-Hyoung Joo

**Affiliations:** 1 Division of Environmental Materials, Honam National Institute of Biological Resources (HNIBR), Mokpo, 58762 Republic of Korea; 2 Department of Environmental Engineering, Division of Civil, Environmental, Mineral Resource and Energy Engineering, Soil Environment Research Center, Jeonbuk National University, 567 Baekje-Daero, Deokjin-Gu, Jeonju, 54896 Republic of Korea

**Keywords:** Environmental biotechnology, Environmental sciences

## Abstract

In this study, the potential of *Chlorella sorokiniana* JD1-1 for biodiesel production was evaluated using domestic wastewater (DWW) as a diluent for locally-generated livestock wastewater (LWW). This strategy aimed to provide sustainable wastewater treatment, reduce environmental impacts, enhance cost-effectiveness, and promote biodiesel production. LWW was diluted with tap water and DWW at ratios of 75%, 50%, and 25% (v/v), and the effects on microalgal growth, nutrient removal efficiency, and lipid yield were evaluated. Although the maximum biomass concentration was observed in the artificial growth medium (BG-11) (1170 mg L^−1^), 75% dilution using tap water (610 mg L^−1^) and DWW (780 mg L^−1^) yielded results comparable to the exclusive use of DWW (820 mg L^−1^), suggesting a potential for substitution. Total nitrogen (TN) removal rates were consistently high under all conditions, particularly in samples with higher concentrations of LWW. Conversely, total phosphorus (TP) concentrations decreased under most conditions, although some displayed large increases. Further studies are necessary to optimize the nutrient balance while maintaining economic feasibility and maximizing biodiesel production.

## Introduction

Wastewater, such as domestic wastewater (DWW) and livestock wastewater (LWW), affects the aquatic environment. For example, the over-release of nitrogen and phosphorus causes eutrophication, leading to harmful algal blooms^1^. Microalgae-based wastewater treatment is a process in which microalgae are grown using wastewater as a source of nutrients, and is called bioremediation or phycoremediation^2^. One benefit of wastewater-based microalgal cultivation is that it can help reduce the environmental impacts of wastewater by removing pollutants and nutrients that can cause eutrophication and other negative effects. This process is not only used for wastewater treatment but also for the production of biofuels, animal feed, and other useful products^3^. In addition, microalgae can be applied in an energy-saving and cost-effective manner, suggesting that this process is an attractive option for developing nations as well^4^.

The isolation, screening, and application of indigenous microorganisms are important for overcoming regional environmental problems caused by wastewater pollution. This is because they have already adapted to the local environment and may be more effective in degrading nutrients than non-indigenous microalgae^5^. Qu et al. used the newly isolated indigenous strain *Parachlorella kessleri* from rivers to treat locally occurring swine wastewater with carbohydrate production^6^. Pandey et al. applied a screening procedure to obtain indigenous native microalgae for the treatment of dairy effluent and lipid accumulation^7^. We previously isolated the indigenous microalga *Chlorella sorokiniana* JD1-1 from the coast of Jindo Island, South Korea, which can treat both domestic and livestock wastewater effectively^8^.

Livestock wastewater, which contains high levels of nutrients, organic matter, and turbidity, should be diluted before use in microalgal cultivation^9^. The dilution method to treat wastewater using diluting materials, such as distilled water^9^, tap water^10^, and microalgal growth medium^11^, is now widely accepted by the research community. Appropriate dilution depends on the type of microalgae being cultivated and the specific properties of the wastewater^12^. The optimized dilution rate can reduce the influence of contamination and help the growth of microalgae-grazing microorganisms for biomass production^13^. One alternative option to dilution is mixed wastewater treatment, which combines multiple wastewater sources and provides a synergistic approach to improving nutrient removal efficiency and production of biomass. The mixture has the potential to produce a balanced nutrient profile, which is essential for optimal microalgal growth. The mixture of high-strength wastewater with less-strength wastewater is considered an attractive option to support microalgal growth by increasing the transmission of light to enhance photosynthesis efficiency^14^. Furthermore, using mixed wastewater can increase biomass yield, making it a more cost-effective option to single-source wastewater treatment^15^. Several studies have shown that mixed wastewater treatment increase biomass productivity, nutrient removal efficiency, and biodiesel production^16, 17^. Therefore, investigating the potential of mixed wastewater in microalgae cultivation is essential for sustainable wastewater treatment and biodiesel production. Additionally, the lipid profile of microalgae grown on mixed wastewater is expected to improve biodiesel quality depending on nutrient or component sources^18^. However, finding suitable diluting strategies with other kinds of wastewater is still challenging, so investigating suitable sources for simultaneous biomass production and wastewater treatment is important.

DWW has the potential to be used as a diluting medium for LWW because it has lower turbidity and nutrient loading than LWW. Therefore, we suggest simultaneous treatment with DWW as a diluting medium for LWW. Several dilution ratios (25%, 50%, and 75%) were compared with single applications of growth medium (BG-11), DWW, and LWW. We also compared tap water with DWW to develop a realistic application of the suggested treatment.

## Materials and method

### Preparation of microalgae inoculation and wastewater

*Chlorella sorokiniana* JD1-1 (OP322968) was selected for wastewater treatment because it has been successfully applied to both DWW and LWW^8^. The isolated strain was continuously cultivated in a sterile BG-11 freshwater solution (C3061, Sigma-Aldrich, USA) at room temperature under light irradiation ranging from 50 to 100 µmol m^−2^ s^−1^.

DWW and LWW samples were collected from a local wastewater treatment plant in City M, South Korea. The basic characteristics of the samples are listed in Table 1. The dominant nitrogen species in each source are nitrate (242.8 ± 8.2 mg NO_3_-N L^-1^ in BG-11 and 45.6 ± 5.0 mg NO_3_-N L^-1^ in DWW) and ammonia (1812.0 ± 10.6 mg NH_3_-N L^-1 ^in LWW).

**Table 1:** Basic characteristics of the growth medium (BG-11), domestic wastewater (DWW), and livestock wastewater (LWW) used to cultivate microalgae.

Sources	pH	TOC (mg L^-1^)	TN (mg L^-1^)	TP (mg L^-1^)
BG-11	6.77	11.9 ± 2.5	242.7 ± 3.3	3.5 ± 0.1
DWW	9.05	6.5 ± 0.5	50.2 ± 0.7	2.3 ± 0.1
LWW	9.47	750.8 ± 32.9	1804.5 ± 6.4	3.7 ± 0.3

*TOC* total organic carbon, *TN* total nitrogen, *TP* total phosphorous.

### Experimental set-up for batch cultivation

A glass bubble column photobioreactor with a working volume of 3 L was used for batch cultivation of microalgae. Continuous aeration was performed at the bottom of the reactor using an air supplier at a rate of 1 L/min. To initiate the batch cultivation, 10% of the seed culture was prepared and the rest was filled with each source for cultivation. The dilution ratios of DWW to LWW (D/L) were 75%, 50%, and 25%. Dilution with tap water (Tap water to LWW; T/L) was also performed at 75%, 50%, and 25% with three types of control tests (control 1: BG-11, control 2: DWW, and control 3: LWW). The detailed experimental procedures are listed in Table 2.

**Table 2:** Experimental set-up in the dilution and cultivation of microalgae.

Experimental Label	Source	Source to LWW ratio (v/v)
Control 1	BG-11	–
Control 2	DWW	
Control 3	LWW	
T/L 75%	Tap water	3:1
T/L 50%		1:1
T/L 25%		1:3
D/L 75%	DWW	3:1
D/L 50%		1:1
D/L 25%		1:3

After the last day of cultivation, all samples were collected and centrifuged at 4500 *rpm* for 20 min. The pellets were lyophilized using a freeze dryer (Freezone, Labconco, USA) for 3 d. The freeze-dried biomass was used as a source for lipid and biodiesel (FAME) extraction.

### Analytical procedures

Samples (50–100 mL) were collected at defined time intervals for analysis. First, the pH value was analyzed using a pH meter (Orion Star™ A211, Thermo scientific, USA). The samples were then vacuum-filtered through a pre-weighed GF/C filter paper (47 mm, Whatman, USA) to analyze the biomass based on the procedures in Standards Methods^19^. The productivity of microalgal biomass was calculated as follows^20^:

1 $${P}_{Biomass} (\mathrm{g}\ {\mathrm{L}}^{-1} {\mathrm{d}}^{-1}) = ({\mathrm{C}}_{2} -{\mathrm{C}}_{1} ) / ({\mathrm{t}}_{2} - {\mathrm{t}}_{1})$$

where P_biomass_ is the biomass productivity (g L^-1^d^-1^), C_1_ and C_2_ are the concentrations of biomass at times t_1_ and t_2_.

Water analysis was conducted using the filtrates. Filtrates were filtered again using a 0.45 µm polyvinylidene fluoride (PVDF) syringe filter for total nitrogen (TN) and total phosphorus (TP) analysis. The TN values were analyzed using a TN analyzer (TNM-L, Shimadzu, Japan) equipped with a Total organic carbon (TOC) analyzer (TOC-LCPH, Shimadzu, Japan). The TP concentration was analyzed using water test kits (HS-TP-H and HS-TP-L, Humas, Korea) according to the manufacturer’s instructions.

### Extraction and analysis of lipid and biodiesel

#### Lipid extraction and transesterification

The modified lipid extraction method suggested by Bligh and Dyer^21^ was used for lipid extraction, using the harvested microalgal biomass^8^. Briefly, 1 mL chloroform, 2 mL methanol, and 0.8 mL of deionized (DI) water were added to the lyophilized biomass (0.5 g). Next, 1 mL chloroform was added and the tubes were vortexed for 2 min. The tubes were vortexed for an additional 30 s following 1 mL of DI water added. The vortexed solution was then sonicated in an ultrasonication bath (UCP-10; Vision Science, Korea) for 10 min. To separate the extract layers, the suspensions were centrifuged at 4150 × *g* for 10 min. The chloroform-containing lipid layers were collected and placed in pre-measured vials. Using a heating block set to 60 ± 5 °C, the solvents were evaporated, weighed, and the lipid contents (w/w, %) calculated.

Lipid transesterification was performed according to Carreau and Dubacq^22^ to evaluate the biodiesel (fatty acid methyl ester; FAME) profile. Briefly, 2 mL of 1% sodium hydroxide (NaOH) based on methanol was added to vials containing residual lipids, and the vials were heated at 60 ± 5 °C for 30 min. Next, 2 mL of 5% hydrochloric acid (HCl) based on methanol was added and the samples heated at 60 ± 5 °C for 30 min. Finally, 2 mL of n-hexane was added to separate the FAME layers in the extracts, and the FAME-containing hexane layers were collected in 2 mL vials and used for FAME profile analysis.

#### Gas chromatographic analysis of biodiesel profiles

The FAME composition of the transesterified products was analyzed using a gas chromatograph (Trace 1310, Thermo Scientific, USA), equipped with a flame ionization detector (FID). Here, 1 µL of the sample was injected into a DB-fastFAME capillary column (20 m × 0.18 mm × 0.2 µm, Agilent, USA). High-purity (99.999%) nitrogen gas was used as the carrier gas at a constant pressure of 193.05 kPa. The split ratios and temperatures of the detector and injector were 1:100, 280, and 250 °C, respectively. The oven temperature was controlled by a programmed ramped temperature as follows: an initial temperature of 80 °C for 0.5 min, increased to 175 °C at a rate of 65 °C min^−1^, and then increased to 185 °C at a rate of 10 °C min^−1^ (hold 0.5 min), 230 °C at a rate of 7 °C min^−1^ (overall retention time was 20 min).

#### Fuel properties of biodiesel

Based on the composition and saturation of FAME, various fuel properties were calculated using empirical equations: saponification value (SV), iodine value (IV), cetane number (CN), long-chain saturated factor (LCSF), cold filter plugging point (CFPP), cloud point (CP), pour point (PP), kinematic viscosity (at 40 °C, n), density (at 20 °C, r), and higher heating value (HHV). Detailed empirical equations were introduced in our previous study^23^.

## Results and discussion

### Biomass production

**Figure 1:**
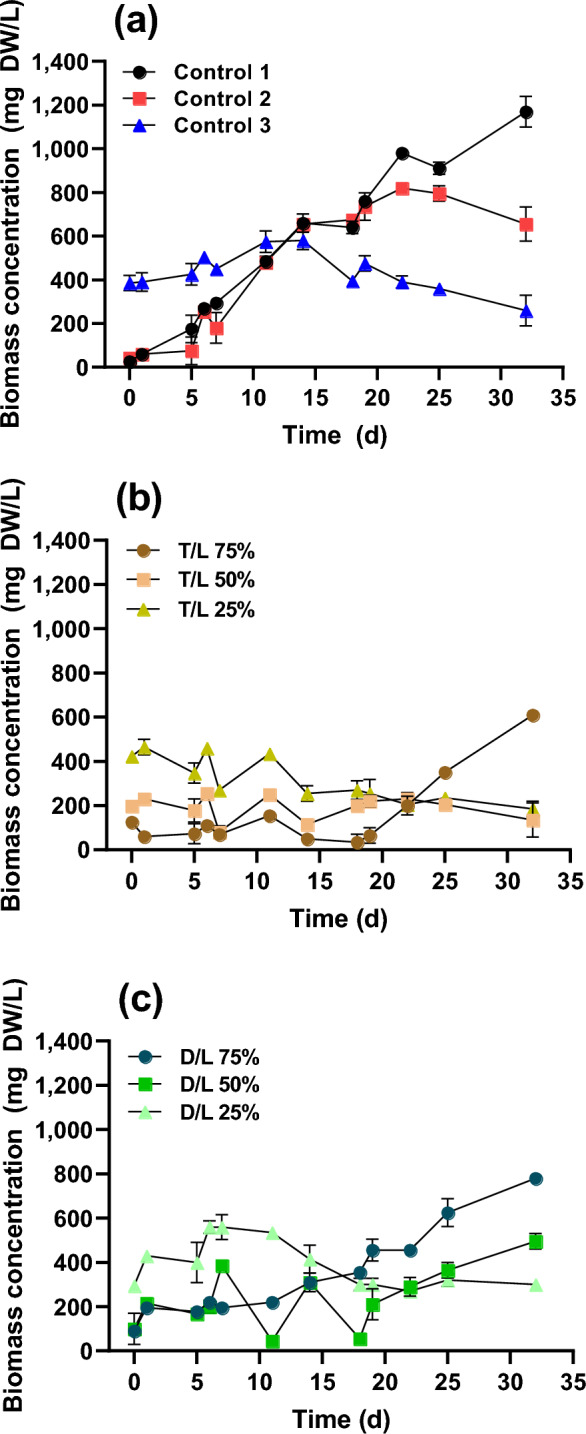
Effects of dilution ratio on the biomass concentration of the microalgae over time. (**a**) Biomass concentration changes in control 1 (BG-11), control 2 (domestic wastewater; DWW), and control 3 (livestock wastewater; LWW). (**b**) Biomass concentration changes for T/L dilution ratios of 75%, 50%, and 25%. (**c**) Biomass concentration changes for D/L dilution ratios of 75%, 50%, and 25%. T/L and D/L indicate the dilution of tap water to LWW and DWW to LWW (v/v), respectively.

Figure 1 shows changes in biomass concentration across different cultivation periods. In the case of BG-11 (control 1), the biomass continuously increased for up to 32 d with a maximum dry biomass concentration of 1170 ± 70.1 mg L^−1^. Cultivation using only DWW (control 2) saw a lag phase of 22 d followed by an increase up to 820 ± 0.0 mg L^−1^, while that of LWW (control 3) only increased up to 195 ± 7.1 mg L^−1^ over 14 d. No significant change in the T/L dilution was observed for 21 d, however, after that, the biomass of T/L 75% began to increase and eventually reached 610 ± 14.1 mg L^−1^. In the case of D/L dilution, D/L 25% showed a tendency to decrease continuously after growth up to 550 ± 56.6 mg L^−1^ for 7 d. The biomass in the 50% D/L treatment began to increase after 20 d and reached 495 ± 35 mg L^−1^ at 32 d. In the case of D/L 75%, the biomass continuously increased after cultivation and reached 780 ± 21.2 mg L^−1^ at 32 d. In our previous studies on the simple application of *C. sorokiniana* JD1-1 to treat DWW and LWW, this strain grew to 875 mg L^−1^ (BG-11), 640–1,050 mg L^-1^ (DWW), and 580 mg L^−1^ (tenfold diluted LWW)^8^. The treatment of T/L 75% and D/L 75% corresponds to the findings of previous studies because the maximum concentration of biomass was 610 ± 14.1 (T/L 75%) and 780 ± 21.2 mg L^−1^ (D/L 75%), respectively.

Based on the concentration of biomass, calculated biomass productivities were 35.75 mg L^−1^ d^−1^ (BG-11), 35.32 mg L^−1^ d^−1^ (DWW), and 13.93 mg L^−1^ d^−1^ (LWW), which were similar to findings from previous studies, which reported 47.6 mg L^−1^ d^−1^ (BG-11) and 35.3–57.0 mg L^−1^ d^−1^ (DWW)^8^. The optimal N/P is usually considered to be 16:1, but some cases have reported higher ratios for microalgae cultivation, such as 22:1 in livestock wastewater^24^, and 190:1 in landfill leachate^25^. In this study, the N/P ratios were calculated as 22:1 for DWW and 490:1 for LWW, with biomass productivity ranging from 12.34 to 18.59 mg L^−1^ d^−1^. By simultaneously treating DWW and LWW, the N/P ratio could be reduced from 490:1 (LWW) to 98:1 (with 25% dilution) and 320:1 (with 75% dilution). Despite of this reduction, the ratio remains high, suggesting that additional phosphorus sources are required for optimal microalgae growth. For economic feasibility, sourcing phosphorus-rich waste resources such as agriculture, sewage, and domestic wastewater^26^ for diluting material would be beneficial. Furthermore, optimizing the N/P ratio through either dilution or continuous operation is essential for enhancing LWW treatment.

### Nutrient removal

**Figure 2:**
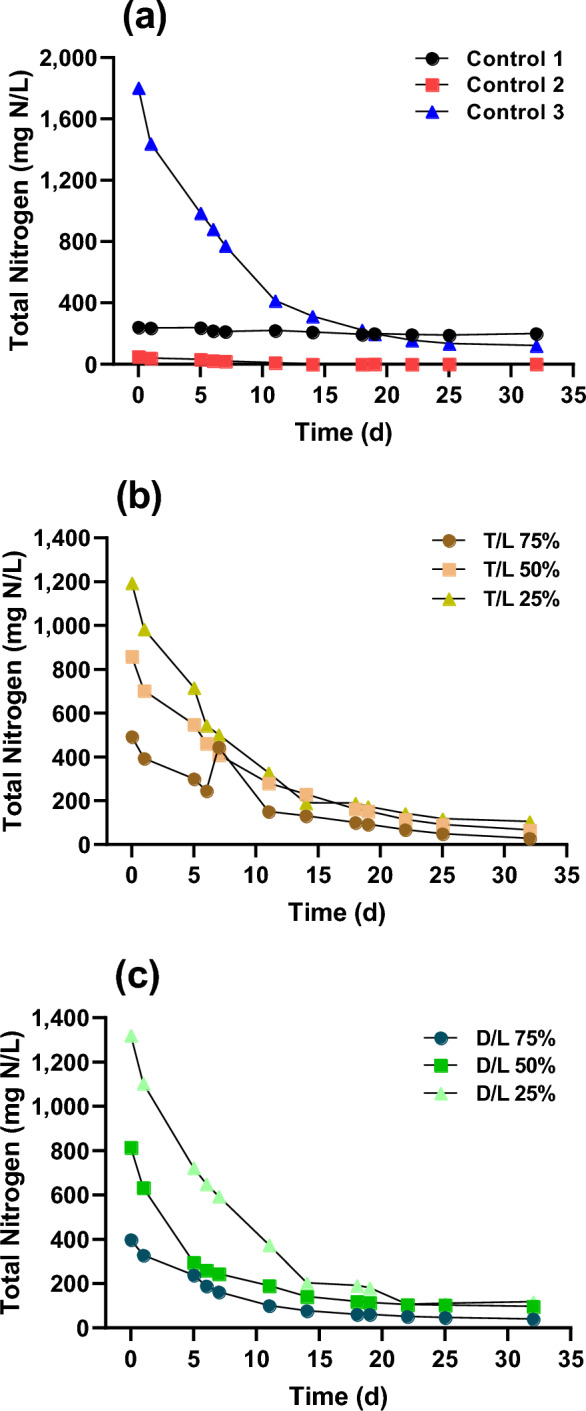
Total nitrogen (TN) concentration under different dilution ratios during microalgae cultivation. (**a**) TN concentration changes in control 1 (BG-11), control 2 (domestic wastewater; DWW), and control 3 (livestock wastewater; LWW). (**b**) TN concentration changes for T/L dilution ratios of 75%, 50%, and 25%. (**c**) TN concentration changes for D/L dilution ratios of 75%, 50%, and 25%. T/L and D/L represent the dilution of tap water to LWW and DWW to LWW (v/v), respectively.

In general, The TN values in the primary- and secondary-treated DWW and LWW were 20–80 mg L^−1^, 5–30 mg L^−1^, and 10–1000 mg L^−1^, respectively. The TP value ranges were 3–7 mg L^−1^, 0.2–3 mg L^−1^, and 9–110 mg L^−1^, respectively (Wang et al. 2017). In this study, the TN values for DWW and LWW were within the general range, at 20.2 mg L^−1^ (DWW) and 1804.5 mg L^−1^ (LWW), respectively. In contrast, the TP values were relatively low at 3.7 mg L^−1^. Figure 2 shows the TN concentration as a function of cultivation time under each experimental condition. In control 1 (BG-11), nutrient removal was relatively low (17.2%), whereas significant degradation was observed under all other experimental conditions. In the case of controls 2 (DWW) and 3 (LWW), 95.0% and 93.2% of the TN were removed, respectively. For the various dilution ratios, the initial TN concentrations and removal efficiencies were as follows: for T/L 75%, the initial concentration was 492.6 mg L^-1^ with a removal efficiency of 94.1%; for D/L 75%, the initial concentration was 398.3 mg L^−1^ with a removal efficiency of 89.8%. For T/L 50%, the initial concentration was 859.8 mg L^−1^ with a removal efficiency of 92.2%, and for D/L 50%, it was 815.0 mg L^−1^ with an 88.0% removal efficiency. Lastly, for T/L 25%, the initial concentration was 1194.8 mg L^−1^ with a removal efficiency of 91.2%, while for D/L 25%, it was 1321.0 mg L^−1^ with a removal efficiency of 91.0%.

There was no significant difference in the medium source. However, the TN removal rate was high when the source had a high initial TN concentration. In particular, higher TN removal was detected in LWW-containing samples (control 3, T/L 25%, and 25% D/L). Microalgae including *Chlorella* spp. selectively absorb NH_4_ via the NH_3_ channel and assimilate NO_3_ and NO_2_ using nitrogen transporters^27^. This capability enables efficient treatment of various nitrogen species in wastewater and allows higher loading rate tolerances. In addition, in the application of LWW treatment using microalgae, the ammonia removal efficiency can be enhanced by aeration for growth through volatilization by stripping and by the metabolism of microalgae^28^. Volatilization for ammonia removal is facilitated under weak alkaline conditions in a pH range of 8 to 10 ^29, 30^. On the other hand, a pH range of 7 to 9 is optimal for microalgal growth^31^. The pH range observed in this study was 8.5 to 9.5, which is conducive both for an optimized CO_2_ supply for microalgal growth and ammonia volatilization.

**Figure 3:**
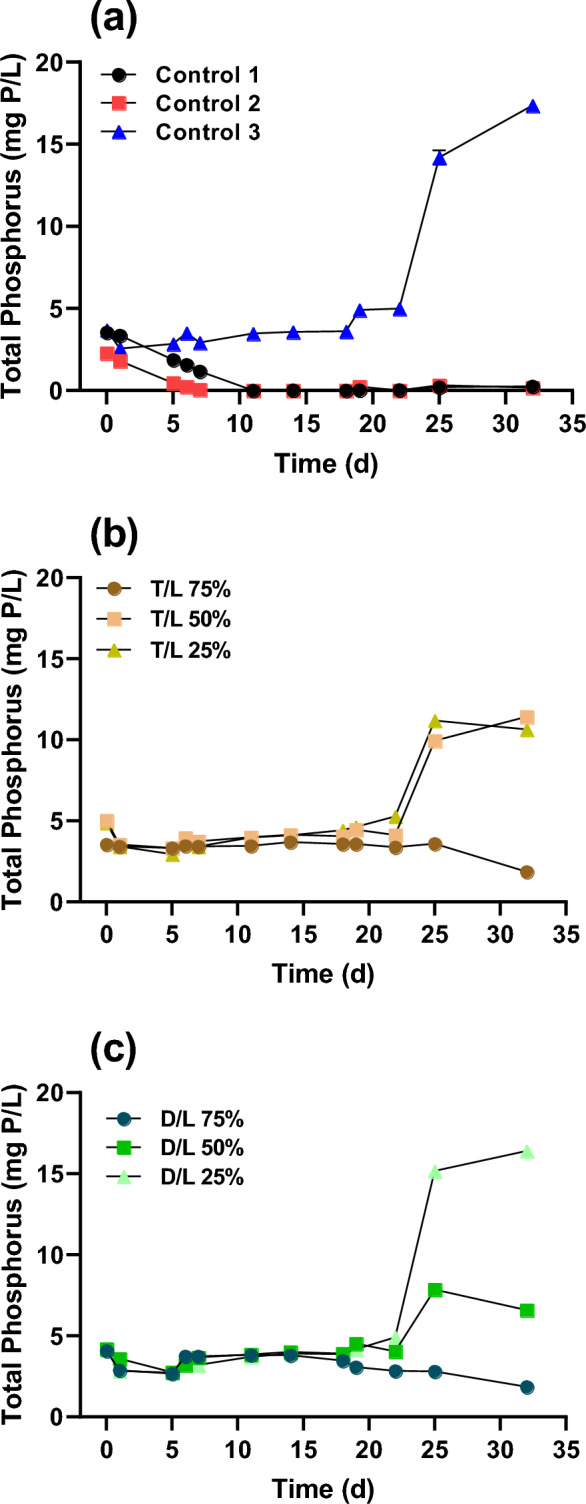
Total phosphorus (TP) concentration under different dilution ratios during microalgae cultivation. (**a**) TN concentration changes in control 1 (BG-11), control 2 (domestic wastewater; DWW), and control 3 (livestock wastewater; LWW). (**b**) TN concentration changes for T/L dilution ratios of 75%, 50%, and 25%. (**c**) TN concentration changes for D/L dilution ratios of 75%, 50%, and 25%. T/L and D/L represent the dilution of tap water to LWW and DWW to LWW (v/v), respectively.

Figure 3 shows the TP concentration as a function of cultivation time for each experimental condition. TP values in control 1 (BG-11), control 2 (DWW), T/L 75%, and D/L 75% decreased with removal efficiencies of 92.8%, 92.4%, 47.5%, and 54.2%, respectively. In contrast, LWW-dominant groups (control 3, T/L 50%, T/L 25%, and D/L 25%) displayed a noticeable increase in TP concentration during the latter stages of cultivation. Such a rise in TP concentration can often be caused by various external factors in wastewater treatments using microalgae. In particular, changes in pH and dissolved oxygen (DO) levels might precipitate certain phosphorus compounds, leading to sedimentation in the medium^28^. The growth of microalgae occurred simultaneously with the reduction of TP, suggesting that the assimilation of phosphorus by microalgae was efficient in conditions that supported their growth through building of phospholipids, nucleotides, and adenosine triphosphate (ATP)^26^. Nevertheless, the increase of TP when adding higher ratio of LWW was not clearly investigated, so further studies are required.

### Yield of lipids and biodiesel

The total lipid content of each microalgal biomass sample is listed in Table 3. The lipid content of DWW was higher than that of the growth medium (BG-11) and LWW. Lipid contents in the T/L 50%, T/L 25%, and D/L 25% groups were not detected because of the small amount of microalgae harvested. Under the other conditions, T/L 75%, D/L 75%, and D/L 50%, the lipids were extracted at 21.1 ± 0.9, 17.9 ± 0.8, and 24.4 ± 0.0, respectively. Both T/L 75% and D/L 75% were not significantly different from DWW but were improved compared to BG-11. Generally, a lack of nutrients or trace elements can increase the lipid content of microalgae^32^. The produced lipids in this study were similar to that from our previous study in the case of BG-11 (12.3% and 10.0%) while DWW was different from that (7.1–8.3% and 17.9–24.4%)^8^. This was caused by lipid accumulation related to nutrient depletion and stress factors, such as salt. Therefore, the selection of waste resources and their optimization is important, in addition to the selection of microalgae species. On the other hand, Jebali et al. suggested an appropriate dilution rate for the production of biomass and nutrient removal using *Scenedesmus* sp., but there was no significant difference between the dilution rate and lipid content^13^.

**Table 3:** Lipid contents from harvested microalgal biomass.

Exp. Label	Lipid yield (%)
Control 1	12.3 ± 3.6
Control 2	20.7 ± 5.4
Control 3	10.9 ± 6.1
T/L 75%	21.1 ± 0.9
T/L 50%	ND
T/L 25%	ND
D/L 75%	17.9 ± 0.8
D/L 50%	24.4 ± 0.0
D/L 25%	ND

*ND* Not detected.

**Figure 4:**
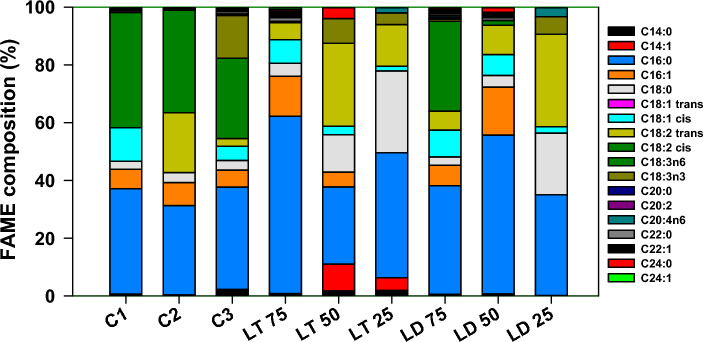
Composition of the Fatty acid methyl ester (FAME) in harvested microalgal biomass.

The fatty acid compositions of the microalgae cultivated under each experimental condition are shown in Fig. 4. The main components of the FAMEs in the control test were palmitic acid (C16:0) and linoleic acid (C18:2). Compared to BG-11, biomass cultivated in DWW had a higher polyunsaturated fatty acid (PUFA) content (56.5% vs. 39.9%), whereas biomass cultivated in LWW had a higher saturated fatty acid (SFA) (41.9% vs. 40.5%) and PUFA (45.9% vs. 39.9%) contents. At T/L 75%, C16:0 (61.4%) was detected with the highest SFA content. For FAMEs in L/D 75%, the ratios of C16:0 and C18:2-trans were relatively high, similar to those in DWW and LWW. The compositions of L/T 75% and L/D 75% also differed in C18:2-cis. Considering that all control tests were of high content, tap water was found to affect the C18:2-cis composition. However, as the dilution ratio changed, changes in C18:2-trans and C18:0 were affected by microalgal growth.

Usually, the FAME composition in microalgal lipids cultivated from wastewater varies depending on several factors, such as the type of microalgae, wastewater composition, and growth conditions. To the best of our knowledge, the effects of the dilution medium on FAME composition have not yet been reported. However, some studies have reported on the dilution ratio of microalgal biodiesel followed by media dilution. Marchão et al.^33^ reported the effects of the dilution rate on the continuous mode of brewery wastewater treatment using *Scenedesmus obliquus*. There, the SFA was more affected by the dilution rate than PUFAs. Jebali et al. compared the dilution rates in the semi-continuous treatment of wastewater using native Tunisian microalgae, and the results showed that biomass productivity increased as the dilution rate increased; however, there was no significant difference in lipid content, and the change in PUFA was highly affected^13^. Kusmayadi et al. discussed the use of dairy wastewater with different dilution factors to produce microalgal biomass, and their results showed that optimal biomass production and nutrient removal were achieved at 50% dilution, whereas the highest lipid content was obtained at 100% wastewater^34^. Vargas-Estrada et al. compared four different dilution factors (0%, 10%, 50%, and 100% wastewater) for the growth of microalgae and found that, while the highest growth and protein content was achieved at a 50% dilution, lower dilution ratios resulted in higher efficiency of biomass utilization for biogas production^35^.

### Fuel properties

The fuel properties of the microalgal FAME are listed in Table 4 and compared with two international standards (ASTM D6751 and EN14214) for IV, CN, CFPP, viscosity, and density. The obtained CN, CFPP, viscosity, and density values were compared to those of ASTM D6751. The target range of CN was 47 or higher; the range of viscosity, density, and CFPP was 1.9–6.0 mm^2^ s^−1^, 0.85–0.9 g cm^−3^, and – 13 to − 5 °C, respectively. All conditions were satisfied for the CN, viscosity, and density, whereas the CFPP varied under each experimental condition. In the case of EN14214, IV was satisfied in all instances with values less than 120, and viscosity ranged from 3.5 to 5.0 mm^2^ s^−1^, except for C3. For CN, the target range was 51 or higher, with values of 47.8 and 48.5 for C2 and C3, respectively. The CFPP ranges were 5 or lower; these values were acceptable in some conditions but not satisfied for LT75, LD50, and LD25. Most fuel properties satisfied international standards. In addition, the viscosity and CN values in the diluted cultures had a positive effect compared to the control test. However, the standards and characteristics of the CFPP values require further optimization because they vary according to the experimental conditions, and the target values differ among countries.

**Table 4:** Fuel properties of the fatty acid methyl esters (FAME) produced by harvested microalgal biomass.

Property	C1	C2	C3	L/T 75%	L/D 75%	L/D 50%	L/D 25%	ASTM D6751	EN14, 214
SFA	40.5	35.3	41.9	68.7	42.8	62.4	56.4		
MUFA	18.5	8.0	10.8	23.0	16.4	23.9	2.2		
PUFA	39.9	56.5	45.9	5.9	39.0	11.8	41.5		
DU	98.3	121	102.6	34.8	94.4	47.5	85.2		
SV	205.5	206.6	205.5	208.0	203.9	208.5	205.4		
IV	89.6	110.6	107.9	32.6	87.7	46.0	87.8		$\le$ 120
CN	52.7	47.8	48.6	65.2	53.3	62.1	53.1	$\ge$ 47	$\ge$ 51
LCSF	6.1	5.5	6.4	11.8	8.1	12.5	14.2		
CFPP	− 2.5	− 0.8	− 3.6	− 20.7	− 8.8	− 22.6	− 28.1	− 13 to − 5	$\le$ 5/$\le$ 20
CP	14.2	11.3	13.7	27.3	14.8	23.9	13.4		
PP	8.6	5.5	8.0	22.8	9.2	19.2	7.7		
APE	91.5	113.2	97.2	20	87.9	30.8	88.5		
BAPE	39.9	57.5	61.2	6.9	40.7	13.4	50.9		
OS	5.5	4.7	5.2	22.6	5.7	12.6	5.7		
HHV	38.9	39.2	38.7	38.3	38.6	38.5	39.5		
υ	3.6	3.5	3.4	3.7	3.6	3.7	3.8	1.9–6.0	3.5–5.0
ρ	0.9	0.9	0.9	0.8	0.9	0.9	0.9	0.85–0.9	

*SFA* Saturated Fatty Acid (%), *MUFA* Mono Unsaturated Fatty Acid (%), *PUFA* Poly Unsaturated Fatty Acid (%), *DU* Degree of Unsaturation, *SV* Saponification Value (mg/g), *IV* Iodine Value, *CN* Cetane number, *LCSF* Long Chain Saturated Factor, *CFPP* Cold Filter Plugging Point (°C), *CP* Cloud Point (°C), *PP* Pour Point (°C), *APE* Allylic Position Equivalent, *BAPE* Bis-Allylic Position Equivalent, *OS* Oxidation Stability (h), *HHV* Higher Heating Value, *υ* Kinematic Viscosity (mm^2^/s), *ρ* Density (g/cm^3^).

Generally, a good composition of FAMEs in microalgal lipids for biodiesel production should include a high percentage of long-chain fatty acids, particularly C16 and C18. Palmitic acid (C16:0) is a common fatty acid found in microalgae and one of the major components of the SFA fraction in microalgal FAMEs. In addition, FAMEs from microalgae containing high ratios of C16:0, C18:1, and C18:2 have high energy content, low viscosity, and improved cold-flow properties, making them suitable for use as biodiesel feedstock^36^.

### Perspective and implication of this study

Microalgal cultivation using LWW diluted with DWW is an economical and effective method for saving water resources. According to the results of this study, DWW was sufficient as a dilution medium instead of tap water, but analysis and consideration are essentially related to the quality and composition of wastewater. First, the ratio of nutrients (nitrogen and phosphorus) should be considered. An optimized nutrient balance can have positive effects on the maximum production of biomass, whereas starved nutrient conditions can affect lipid accumulation. In the use of a diluted medium, the nutrient ratio varied with resources, so that the optimized dilution ratio could be applied based on the purpose of cultivation. The presence of pollutants in wastewater can affect microalgal growth. DWW and LWW contain various pollutants, such as heavy metals, organic compounds, and pathogens. Pollutants in wastewater inhibit growth (photosynthesis)^37^, and increase the generation of reactive oxygen species that cause adverse effects such as lipid peroxidation, protein oxidation, and nucleic acid damage^38^.

Additionally, some bacterial and fungal pathogens and viruses can infect microalgal cells and promote cell lysis, thereby inhibiting biomass production and overall process control^39^. Thus, some researchers have used microalgae as models to assess ecological toxicity^40–42^. Despite their toxicity, some microalgal species can be used for bioremediation through biosorption or consumption of trace metals for enzymatic activity and cell metabolism^40^.

In addition to pathogens, LWW and DWW contain various types of bacteria and microorganisms. Microorganisms present in the culture media can inhibit the growth of microalgae through nutrient competition^43^. This makes it difficult to establish and maintain healthy microalgal cultures. However, some positive effects of the bacterial-microalgae consortium also exist. Organic matter removal, reduced nitrogen loading in high-strength wastewater through nitrification–denitrification, and a supply of CO_2_ for microalgae growth can be expected^44^.

As mentioned earlier, dilution strategies using DWW and LWW have the advantages of reducing the cost of wastewater treatment and enhancing treatment efficiency. However, for improved and realistic applications, an overall techno-economic analysis of media dilution should be performed. Adequate monitoring and management help ensure the sustainability of the process. Our previous study reported the economic feasibility of using wastewater as a growth medium and suggested the possibility of its application as an alternative^45^.

## Conclusions

This study found that microalgal cultivation employing wastewater as a growth medium presents a sustainable and cost-effective strategy for both wastewater treatment and biodiesel production. This study found that diluting livestock wastewater with domestic wastewater is an efficient and economical alternative to tap water. However, further research is required to optimize the growth conditions and biodiesel production of microalgae using different types of wastewater as growth media. In addition, the need for adequate monitoring and management systems to ensure the overall sustainability of the proposed process cannot be overstated.

## Data Availability

The datasets used and/or analysed during the current study available from the corresponding author on reasonable request.
